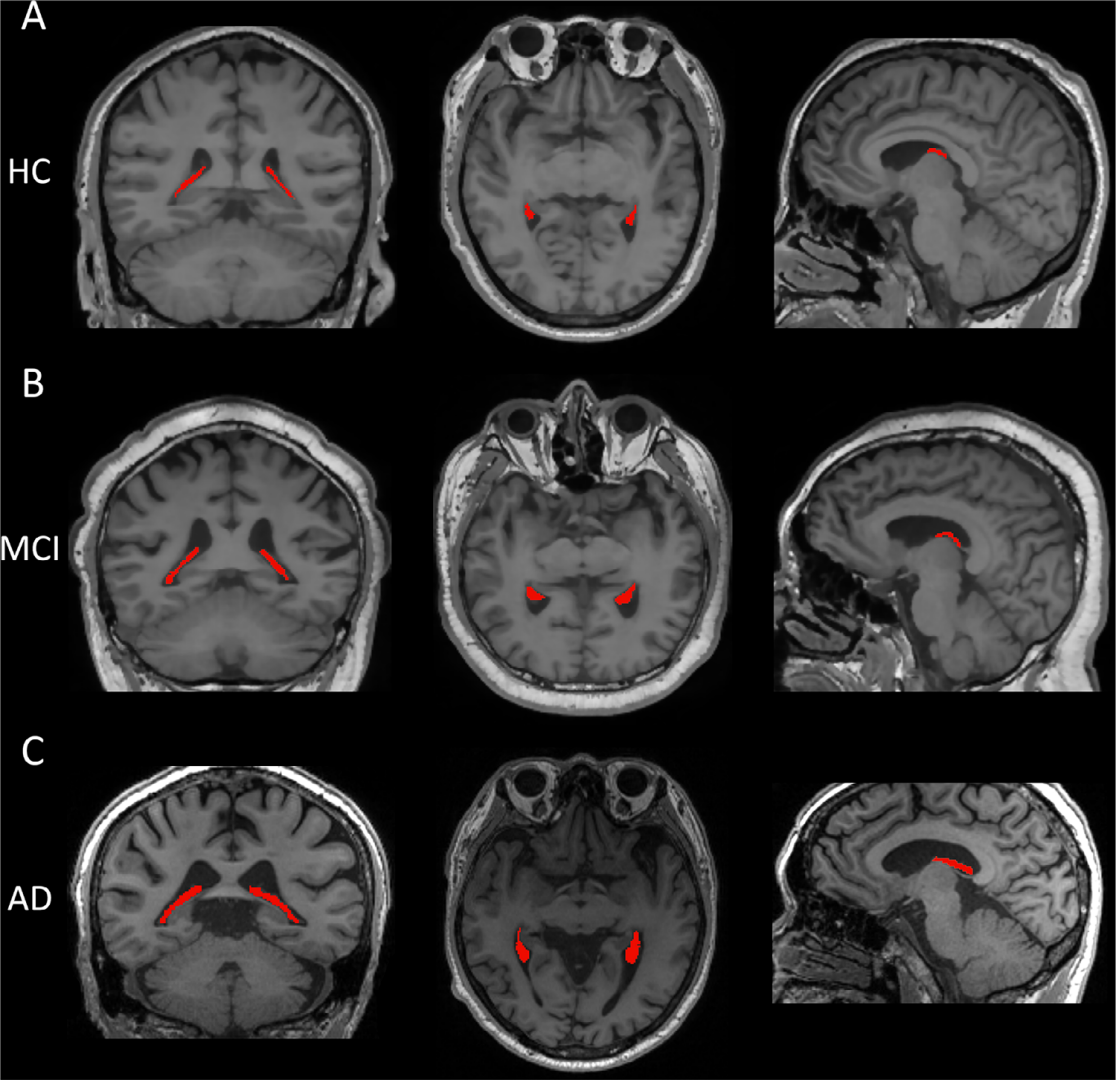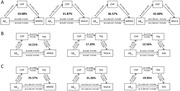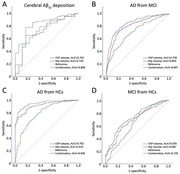# Choroid plexus volume as a novel candidate neuroimaging marker of the Alzheimer's continuum: a prospective cohort study

**DOI:** 10.1002/alz.084867

**Published:** 2025-01-09

**Authors:** Jiwei Jiang, Wenyi Li, Shirui Jiang, Jun Xu

**Affiliations:** ^1^ Beijing Tiantan Hospital, Capital Medical University, Beijing China; ^2^ Beijing Tiantan Hospital, Capital Medical University, Beijing China; ^3^ Beijing Tiantan Hospital, Capital Medical University, Beijing China

## Abstract

**Background:**

The clinical role and potential mechanisms of the choroid plexus (ChP) in Alzheimer's disease (AD) remains unclear.

**Method:**

This prospective cohort study enrolled 607 participants [110 healthy controls (HCs), 269 mild cognitive impairment (MCI), and 228 AD dementia] from the Chinese Imaging, Biomarkers, and Lifestyle study between January 1, 2021, and December 31, 2022. Relationship between ChP volume and the cerebrospinal fluid (CSF) pathological hallmarks (Aβ_42_, Aβ_40_, Aβ_42/40_, tTau, and pTau), neuropsychological tests [Mini‐Mental State Examination (MMSE), Montreal Cognitive Assessment (MoCA), Neuropsychiatric Inventory (NPI), and Activities of Daily Living (ADL) scores], and multimodal neuroimaging measures were analyzed using partial Spearman’s correlation. The mediating effects of ChP volume were examined on the relationship between CSF hallmarks and neuropsychological tests. The ChP volume performance to differentiate the presence/absence of cerebral Aβ_42_ deposition was determined using receiver operating characteristic analysis. Generalized linear mixed‐effects models discerned the association between baseline ChP volume and longitudinal changes in neuropsychological tests in patients on AD continuum.

**Result:**

The participants' mean age was 65.99±8.79 years. Patients with AD dementia exhibited a larger baseline ChP volume than the other participants (P<0.001; Figure 1). ChP volume enlargement correlated with decreased Aβ_42_ and Aβ_40_ levels; lower MMSE and MoCA and higher NPI and ADL scores; lower volume, cortical thickness, and corrected cerebral blood flow in other cognition‐related regions (all P < 0.05). ChP volume alone mediated and ChP–hippocampal volume combined chain mediated the association of CSF Aβ_42_ and Aβ_40_ levels with the MMSE scores (16.91%, 37.15%, 14.25%, and 27.82%, respectively; Figure 2). ChP volume better identified the presence/absence of cerebral Aβ_42_ deposition than hippocampal volume (AUC: 0.762 vs. 0.724; Figure 3). Baseline ChP volume was associated with subsequent decline and faster worsening in the MMSE, MoCA, and ADL scores with 10.03±4.45 months' follow‐up (all P<0.001).

**Conclusion:**

ChP volume is a novel, candidate, non‐invasive neuroimaging marker associated with neurodegenerative changes in Alzheimer’s continuum. It can detect early cerebral Aβ_42_ deposition and predict prognosis in clinical practice.